# Gene editing in monogenic autism spectrum disorder: animal models and gene therapies

**DOI:** 10.3389/fnmol.2022.1043018

**Published:** 2022-12-14

**Authors:** Na Wang, Longbao Lv, Xiaoyi Huang, Mingqin Shi, Youwu Dai, Yuanyuan Wei, Bonan Xu, Chenyang Fu, Haoyu Huang, Hongling Shi, Yun Liu, Xintian Hu, Dongdong Qin

**Affiliations:** ^1^School of Basic Medical Sciences, Yunnan University of Chinese Medicine, Kunming, Yunnan, China; ^2^Key Laboratory of Animal Models and Human Disease Mechanisms of the Chinese Academy of Sciences and Yunnan Province, Kunming Institute of Zoology, Chinese Academy of Sciences, Kunming, Yunnan, China; ^3^Department of Pediatric Rehabilitation Medicine, Kunming Children’s Hospital, Kunming, Yunnan, China; ^4^Department of Rehabilitation Medicine, The Third People’s Hospital of Yunnan Province, Kunming, Yunnan, China

**Keywords:** autism spectrum disorder, gene editing, animal model, pathogenesis, therapy

## Abstract

Autism spectrum disorder (ASD) is a lifelong neurodevelopmental disease, and its diagnosis is dependent on behavioral manifestation, such as impaired reciprocal social interactions, stereotyped repetitive behaviors, as well as restricted interests. However, ASD etiology has eluded researchers to date. In the past decades, based on strong genetic evidence including mutations in a single gene, gene editing technology has become an essential tool for exploring the pathogenetic mechanisms of ASD *via* constructing genetically modified animal models which validates the casual relationship between genetic risk factors and the development of ASD, thus contributing to developing ideal candidates for gene therapies. The present review discusses the progress in gene editing techniques and genetic research, animal models established by gene editing, as well as gene therapies in ASD. Future research should focus on improving the validity of animal models, and reliable DNA diagnostics and accurate prediction of the functional effects of the mutation will likely be equally crucial for the safe application of gene therapies.

## Introduction

Autism spectrum disorder (ASD) is a lifelong neurodevelopmental disorder characterized by deficits in social communication with restricted and repetitive behaviors (Lord et al., 2018). Approximately 1.5% of the world population is affected by ASD (Schaaf et al., 2020), and about 50%–60% of ASD cases have a genetic etiology (Taylor et al., 2020). Despite the obvious challenges associated with the identification of ASD causes, intensive genetic studies have confirmed that ASD has a strong genetic basis and many susceptibility genes have been identified by genetic analysis (Havdahl et al., 2021). For ASD, the development is centered around the monogenic forms (Weuring et al., 2021). In recent decades, many efforts have been put into developing new ASD animal models established by gene-editing techniques which target mutations introduced into the genome, and then the phenotype is characterized to explore gene expression profiles specific to ASD (Fan et al., 2020). Gene-editing techniques including zinc finger nuclease (ZFN; Hamilton et al., 2014), transcription activator-like effector nuclease (TALEN), clustered regularly interspaced short palindromic repeats (CRISPR)/CRISPR-associated protein 9 (Cas9) systems (Zhang et al., 2019), and Cre-loxp systems (Mclellan et al., 2017) can utilize various modifications to achieve rapid and targeted gene editing, such as site-directed mutagenesis, insertion, knockdown, and combinatorial editing. At the same time, novel gene therapies have been developed on the aforementioned basis which may contribute to identifying ideal therapeutic candidates for ASD. Here, we review the progress in gene editing techniques and genetic research, animal models established by gene editing, as well as gene therapies in ASD, highlight key issues in ASD-associated model studies, and attempt to identify potential avenues for the treatment of ASD. This will provide valuable insights into the application of gene editing techniques for exploring the pathogenetic mechanisms and therapies for ASD.

## Progress in Gene Editing Techniques

**Figure 1:**
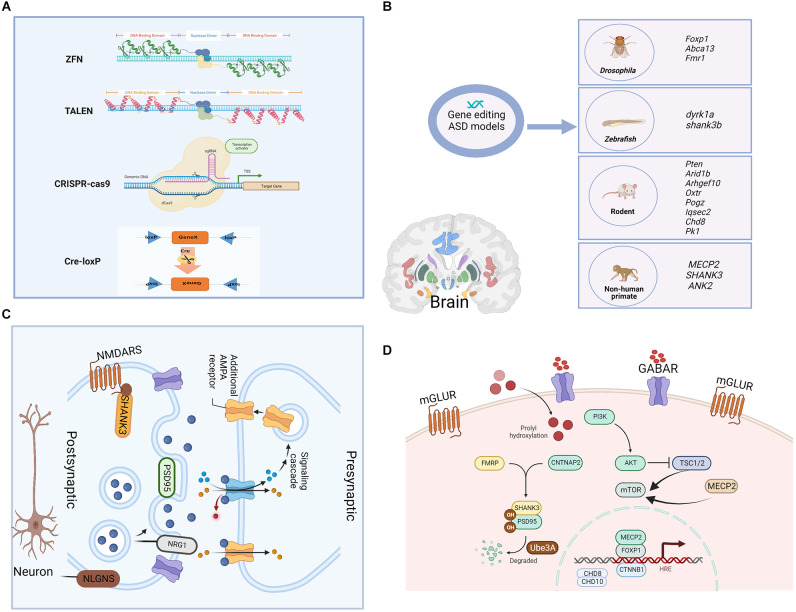
Gene-editing technologies and single gene-edited animal models of ASD as well as the synaptic and cellular functions of modified genes. **(A)** The main genomic editing technologies available at the moment. (a) Zinc Finger Nucleases—ZFNs; (b) Transcription Activator-Like Effector Nucleases—TALENs; (c) CRISPR/Cas9; and (d) Cre-loxP recombinase system. **(B)** Single gene-edited animal models of ASD. Functions of the shown genes: *Foxp1*. forkhead box protein 1, encodes a transcription factor important for the early development of many organ systems, including the brain. *Abca13*: ATP-binding cassette protein A13, is predicted to have the typical structure of full-size ABC proteins, consisting of two transmembrane domains (TMDs). *Fmr1*: fragile X mental retardation 1, is primarily associated with neuro/psychiatric risks. *dyrk1a*: dual specificity tyrosine phosphorylation regulated kinase 1A, is a murine homolog of the Drosophila minibrain gene and has been found to be involved in many biological processes during development and adulthood. *shank3b*: SH3 and multiple ankyrin repeat domains 3, are predicted to enable ionotropic glutamate receptor binding activity and synaptic receptor adaptor activity. *Pten*: phosphatase and tensin homolog, is an essential gene for proper control of cell movement and migration. *Arid1b*: ATT-rich interaction domain 1B, encodes a chromatin remodeling factor, and its haploinsufficiency can cause abnormal gene expression in the brain and induce ASD-like behaviors in mice. *Arhgef10*: Rho guanine nucleotide exchange factor 10, is a known guanine nucleotide exchange factor (GEF) for RhoA with proposed roles in various diseases. *Oxtr*: oxytocin receptor, shows close association with prosocial behavior, and produced numerous pro-social effects through intranasal applications of oxytocin. *Pogz*: pogo transposable element derived with ZNF domain, encodes a multidomain nuclear protein involved in transcriptional regulation and its defective function has been recently associated with a syndromic neurodevelopmental disorder. *Iqsec2*: isoleucine-glutamine motif and Sec7 domain-containing protein 2, is an X-linked gene that is associated with an autism spectrum disorder. *Chd8*: chromodomain helicase DNA binding protein 8, functions in several processes that include transcriptional regulation, epigenetic remodeling, promotion of cell proliferation, and regulation of RNA synthesis. *Pk1*: prokineticin 1, encodes a nuclear receptor that may be a negative regulator of the Wnt/beta-catenin signaling pathway. *MECP2*: methyl-CpG binding protein 2, is an X chromosome-linked protein coding gene encoding the MECP2 protein, which is important for the function of nerve cells and plays a role in maintaining connections (synapses) between neurons. *SHANK3*: SH3 and multiple ankyrin repeat domains 3, play a role in the functioning of synapses, and act as a scaffold that supports the connections between neurons, ensuring that the signals sent by one neuron are received by another. *ANK2*: giant ankyrin 2, plays a role in endocytosis and intracellular protein transport, and is required for synapse stability. **(C)** Synaptic function of modified genes in the ASD model. AMPA, α-amino-3-hydroxy-5-methyl-4-isoxazole propionic acid; PSD95, postsynaptic density 95; NRG1, neuregulin; NMDARS, N-methyl D-aspartate (NMDA) receptors; NLGNS, Neuroligins. **(D)** Cellular function of modified genes in the ASD model. mGLUR, metabotropic glutamate receptors; GABAR, gamma-aminobutyric acid receptor; PI3K, phosphoinositide 3-kinase; TSC1/2, tuberous sclerosis 1/2; mTOR, mammalian target of rapamycin; FMRP: CNTNAP2, contactin associated protein 2; CTNNB1, catenin beta 1.

Gene editing is a genetic engineering technology that can modify, delete, or insert a small piece of DNA at a specific point in the genome of cells and organisms, which holds great promises in the fields of gene detection, gene function regulation, drug research and disease treatment (Cao et al., 2020). Cre-loxp system makes it possible to achieve the precise spatiotemporal control of gene expression by introducing point mutations or insertions/deletions into the host genome. First- and second-generation methods, ZFN and TALEN, respectively, employ nucleases that have both DNA recognition binding domains and DNA cleavage domains (Zhang et al., 2018). The DNA-binding domain in these systems is specially designed to attach to the specific DNA sequence that the DNA nuclease domain cleaves. However, due to their high cost, high off-target likelihood, and complex structure, these first- and second-generation gene editing technologies’ wide range of applications suffer from a low target recognition rate (Huang S. et al., 2021), and now they are replaced by CRISPR/Cas9 which relies on Cas9 and a single guide RNA (sgRNA) to achieve precise cutting to offer ease of design, development and increased efficacy (Kantor et al., 2020). Prior to the introduction of gene editing technologies, natural, physical, or chemical mutagenesis and random insertion of transgenic DNA were the principal approaches used to generate mutations within targeted cells. However, by their nature, none of these approaches could achieve gene editing at specific desired loci, and they were also disadvantaged by random mutagenic events, low efficiency, as well as time-consuming, laborious, and costly (Yang et al., 2017). Due to the development of improved genetic editing systems, the study of the function of selected genes and their relationship with disease-related phenotypes was simplified. Gene editing techniques are powerful tools to model gene–base disorders, as it allows researchers to precisely study the association between genes or genetic variants and the development of an altered phenotype, which helps to explore the pathogenetic mechanisms of ASD. A schematic diagram of the main gene editing techniques is shown in Figure 1A. The detailed pros and cons of the main gene editing techniques are shown in Table 1.

**Table 1:** Pros and cons of different gene editing techniques, and monogenic ASD animal models established by gene editing.

**Technology**	**Number of edited genes**	**Off-target rate**	**Cost**	**Experimental period**	**Pros and cons**	**References**
ZFN	Single or multiple genes	Higher	$25,000	Long (months)	High specificity; high cost; slow speed	Markenscoff-Papadimitriou et al. (2021)
TALEN	Single gene	Lower	$1,000	Short (days)	Simple design; high specificity; weak cytotoxicity; cumbersome assembly process	Kim et al. (2017)
CRISPR/Cas9	Single or multiple genes	Lower	$500	Short (days to weeks)	Short cycle; high efficiency; high chimerism rate; stable reproductive inheritance; large DNA segment knock-in (up to 15Kb long); conditional knockout (up to 4kb long); large knockout (up to 500kb)	Williams et al. (2016), Dhamne et al. (2017), Shibutani et al. (2017), Liu et al. (2018), Horie et al. (2019), Tu et al. (2019), Matsumura et al. (2020), Hoffmann and Spengler (2021), Lee et al. (2021), Mehta et al. (2021), and Ban et al. (2022)
Cre-loxP	Single or multiple genes	Lower	$200	Short (days to weeks)	Spatial specificity; high efficiency; a wide range of applications	Lu et al. (2018)
Cre-loxP	Single or multiple genes	Lower	$200	Short (days to weeks)	Spatial specificity; high efficiency; a wide range of applications	Lu et al. (2018)
**Genes**	**Gene-editing technology**	**Animal**	**Main phenotypical observations**	**References**
ABCA13	RNAi	Drosophila	Increased social space with the closest neighbor and satellite boutons in presynaptic terminals of motor neurons; early onset of evening activity in adult flies	Ueoka et al. (2018)
FMR1	UAS-GAL4 system	Drosophila	Apoptotic cell loss in all adults	Wan et al. (2000)
DYRK1A	TALEN	Zebrafish	Exhibited social disorders that recapitulate the human ASD phenotype	Kim et al. (2017)
SHANK3B	CRISPR/Cas9	Zebrafish	Produced robust ASD-like behaviors and induced alterations in the synaptic proteins homer1 and synapsin levels	Liu C. X. et al. (2018)
PTEN	CRISPR/Cas9	Mice	Decreased dendritic arborization of developing neurons	Williams et al. (2016)
Arid1b	CRISPR/Cas9	Mice	Demonstrated ASD-like behaviors including social disorder, anxiety, and perseverance, as well as body weight loss, impaired motor coordination and hydrocephalus	Shibutani et al. (2017)
Arhgef10	Cre-loxP	Mice	Social interaction impairment, hyperactivity, and decreased depression-like and anxiety-like behavior; increased neurotransmitters including serotonin, norepinephrine, and dopamine in different brain regions; decreased monoamine oxidase A	Lu et al. (2018)
OXTR	CRISPR/cas9	Microtus ochrogaster	Engendered repetitive behaviors, and an impairment in preference for social novelty	Horie et al. (2019)
POGZ (Q1042R)	CRISPR/cas9	Mice	ASD-related behaviors and impaired embryonic cortical neuronal development	Matsumura et al. (2020)
IQSEC2	CRIPSR/cas9	Mice	Abnormal social behaviors relevant to autism	Mehta et al. (2021)
CDH8	CRISPR/ cas9	Mice	Imbalance between excitation and inhibition (E/I balance) in cortical and subcortical circuits	Hoffmann and Spengler (2021)
IQSEC2	CRISPR/ cas9	Mice	Re-expression of IQSEC2 isoform 1 in the mPFC of IQSEC2 KO mice using adeno-associated virus (AAV) improves ASD-behaviors and rescues both synaptic and social deficits	Mehta et al. (2021)
SHANK1 and SHANK3	CRISPR/Cas9	Mice	A low survival rate, severe ASD behavioral impairments, and a strong reduction in the activation of intracellular signaling pathways involving Akt, S6, ERK1/2, and eEF2 during development	Mossa et al. (2021)
PK1(R104Q)	CRISPR/Cas9	Mice	Reduced density of glutamatergic synapses in the hippocampus; impaired interaction with REST; spatial memory deficit and novel object recognition deficit	Ban et al. (2022)
MECP*2*	Lentivirus-based transgenic	Monkeys	ASD-like behaviors, including higher frequency of repetitive cycle movement and increased stress response, and shows transgenic germline transmission	Liu et al. (2016)
MECP2	TALEN	Monkeys	Similar physiological, behavioral, and structural abnormalities of clinical RTT-manifestations, and similar immune gene dysregulation	Chen et al. (2017)
SHANK3	CRISPR/Cas9	Monkeys	Increased sleep disturbances, motor disorders and repetitive behaviors, as well as social and learning disabilities	Zhou et al. (2019)
SHANK3	CRISPR/Cas9	Monkeys	Core behavioral and brain network impairments	Tu et al. (2019)
MECP2	CRISPR/Cas9	Monkeys	Core ASD-like phenotypes	Wu S. et al. (2021)
ANK2	CRISPR/Cas9	Monkeys	Did not cause core ASD-like behaviors, but resulted in drastic changes in brain structure	Qin et al. (2021)

With the continuous development of high-throughput sequencing, such as genome, transcriptome, and proteome, effective methods have been developed for analysis, identification, verification, and application of biomarkers for ASD. When combined with animal models, they will also finally provide insights into the pathogenesis, accurate diagnosis, and development of novel drugs for ASD.

## Genetic Research on ASD

Like many other neuropsychiatric disorders, ASD is a high-risk disorder for genetic alterations that affect the expression or function of proteins involved in the formation and maintenance of synapses, or in chromatin remodeling. Genetic abnormalities in ASD can be divided into three categories: single gene mutations (e.g., SH3 and multiple ankyrin repeat domains 3 (*Shank3*), chromodomains helicase DNA-binding (Chd), methyl-CpG binding protein 2 (*MeCP2*), contactin associated protein (*Cntnap)*, fragile X messenger ribonucleoprotein (*Fmr*), ubiquitin protein ligase E3A (*Ube3a*), TSC complex subunit 1 (*Tsc1*), TSC complex subunit 2 (*Tsc2*) and oxytocin receptor (*Oxtr*); polygenic mutations; and copy number variants including chromosomal duplication, large deletions, inversions, and translocations (Mashayekhi et al., 2021). In this review, we will focus on ASD models based on single gene mutations, which are summarized in Table 1. Most genetic findings implicated in the pathogenesis of ASD to date are inconsistent. However, there are some examples of positive replications. Here, we will review most of the genes that have been consistently related to ASD.

SHANK proteins are multidomain scaffold proteins of the postsynaptic density, which connect neurotransmitter receptors, ion channels, and other membrane proteins to the actin cytoskeleton and G-protein-coupled signaling pathways. They are fundamental to synapse formation and dendritic spine maturation. Gene variants of the *Shank* family are significantly associated with the development of ASD (Ma et al., 2021). When mutations occur in the *Shank* gene, it leads to ASD characterized by impairments in social interaction and communication, and restricted behavioral patterns and interests. Moreover, *Shank* gene family members play important roles in postsynaptic organization by forming complexes containing postsynaptic receptors, ion channels, neurexins (NRXNs), and neuroligins (NLGNs; Medina et al., 2018). SHANK3 and SHANK2, involved in dendritic spine morphogenesis during neuron development, are essential scaffolding proteins found specifically in the postsynaptic density (PSD) of excitatory neurons. Mutations in these two genes can lead to impaired motor coordination, repetitive behavior, and altered social interactions (Woike et al., 2022). In *Shank2*-deficient mice, synapses, chromatin, ribosomes, mitochondria, GABA (γ-aminobutyric acid) neurons, and oligodendrocytes may show alterations and abnormal functions (Lee D. K. et al., 2021). Mutations in the pre-and post-synaptic adhesion proteins NRXNs and NLGNs have been associated with ASD (Wang et al., 2018). In addition, NLGNs have been shown to interact with postsynaptic scaffolding protein, whereas SHANK3 is required for glutamatergic synaptic maturation. NRXNs act as ligands for NLGNs, ranging from transsynaptic interactions between NRXNs and NLGNs to postsynaptic density complex, including SHANK3 scaffold proteins (Yamagata et al., 2018). Eukaryotic translation initiation factor 4E binding protein 2 (EIF4E-BP2) acts as a regulator of synapse activity and neuronal stem cell renewal by its ability to repress translation initiation, which is involved in synaptic plasticity, learning, and memory formation. The absence of the EIF4E-BP2 leads to the overexpression of NLGN-class cell adhesion molecules. Contactins are axon-associated cell adhesion molecules, play an important role in neuronal network formation and plasticity. Moreover, they are glycosylphosphatidylinositol-anchored neuronal membrane proteins that participate in axon connections in the developing nervous system. Especially, CNTN4 (contactin 4) is an immunoglobulin superfamily domain cell adhesion molecules (IgCAMs) protein with important functions associated with fear memory and is related to altered neuronal morphology and synaptic plasticity of hippocampal neurons (Oguro-Ando et al., 2021). Extensive studies have found that the contactin-associated protein-like (CNTNAP) family played key roles in synaptic development and social behavior. For example, CNTNAP3 has been found to interact with synaptic adhesion proteins (neuroglia 1 and 2), as well as scaffold proteins (PSD95 and gephyrin; Tong et al., 2019). Mice with *Cntnap2* mutations showed a reduced frequency of prefrontal projection neural clusters in the cingulate cortex, and this finding revealed a possible selective modulation of *Cntnap2* by integrating prefrontal regional connectivity (Liska et al., 2018). *MeCP2* is an X chromosome-linked gene that encodes the MECP2 protein, which is widely expressed in the developing and mature brain, acts as a key epigenetic regulator of gene expression, and is essential for neural function (Lu et al., 2020). MECP2 was first found to function as a transcriptional repressor. It binds to the CpG island of methylated DNA and recruits co-repressors, such as the histone deacetylase (HDAC) complex, for transcriptional repression. A number of genetic studies have found that *MeCP2* mutation affects synaptic development and neural circuit connectivity in Rett syndrome (RTT). RTT shares some common features with ASD, including motor function deficits, cognitive impairment, and other symptoms associated with mental retardation. Interestingly, *MeCP2* has been reported to bind and repress long genes involved in neuronal differentiation and modulation of neuronal function.

Several members of the CHD protein family are involved in synaptic adhesion, axon growth, and guidance which are critical for brain development. *Chd8, Chd9*, and *Chd11* are three of the most widely studied candidate genes in the field of ASD genetics (Wang et al., 2019). In particular, *Chd8* encodes a neurite outgrowth-regulating membrane protein cadherin-8, and it is one of the most frequently mutated genes in ASD patients. Its mutation causes an imbalance between excitatory and inhibitory neurotransmitters and has been linked to ASD and learning disability (Suetterlin et al., 2018). The oxytocin receptor (*Oxtr*) gene showed a close association with prosocial behavior and produced numerous pro-social effects through intranasal applications of oxytocin (Striepens et al., 2011). During brain development, changes of gene methylation occurred in response to environmental changes in the ASD. For example, the *Oxtr* gene encoding the oxytocin receptor is significantly hypermethylated in the peripheral blood cells and in the temporal cortex of ASD patients, highlighting the reduced oxytocin signaling in ASD. Disconnected (disco)-interacting protein 2 homolog A (DIP2A) protein participates in the synthesis of acetylation-enzyme A (Ac-CoA), which is mainly expressed in brain regions enriched with pyramidal neurons (Ma et al., 2019). The ATT-rich interaction domain 1B (ARID1B) protein encodes a chromatin remodeling factor, and its haploinsufficiency can cause abnormal gene expression in the brain and induce ASD-like behaviors in mice (Shibutani et al., 2017). Engrailed-2 (En2) is a transcription factor that promotes neuronal differentiation of GABAergic neurons in the basal ganglia *via* TrkB (tropomyosin receptor kinase B)-dependent BDNF (brain-derived neurotrophic factor) signaling (Carratala-Marco et al., 2018).

T-box brain transcription factor 1 (TBR1) is a homodimer involved in axonal projection development and neuronal activation in the amygdala, which plays a critical role in the regulation of social interaction and auditory communication. TBR1 interacts with the forkhead box protein P2 (FOXP2) to cause speech and language disorders (Medvedeva et al., 2019). The *Fmr1* gene encodes fragile X mental retardation protein (FMRP), a polyribosome-associated protein playing an important role in protein translation. FMRP targets chromatin modifier genes essential for encoding postsynaptic density proteins (Goin-Kochel et al., 2017). It has been reported that both loss of function and over-expression of UBE3A reduce dendritic branching, suggesting that an appropriate expression level of *Ube3a* is necessary for normal dendritic pattern (Dindot et al., 2008). ASD-associated proteins such as FMR1 (Jansen et al., 2017), pogo transposable element derived with ZNF domain (POGZ; Zhao et al., 2019), TSC complex subunit 1 (TSC1)/TSC complex subunit 2 (TSC2; Martin et al., 2020), and phosphatase and tensin homolog (PTEN; Grencewicz et al., 2021), are all involved in the regulation of protein synthesis. Additional risk genes for ASD include dual specificity tyrosine phosphorylation-regulated kinase 1A *(Dyrk1a)* (Earl et al., 2017), kinesin family member 1 *(Kif1)* (Huang Y. et al., 2021, and trafficking protein particle complex subunit 9 *(Trappc9)* (Cogne et al., 2019). Since the etiology for a substantial portion of ASD remains unknown, these genes may only represent the tip of the “heritable ASD” iceberg.

## ASD Models Established by Gene Editing

Current genetic ASD animal models reported in previous studies include transgenic, knock-in, and gene mutations in *Drosophila*, zebrafish, rodents, and nonhuman primates (Figure 1B), which are based on single gene mutations mainly in the following genes: synaptogenesis and synaptic plasticity-related genes (*Shank3*, *Nrnx1*, *Nlgns*, *Nlgn4*, *Dat*, *Mglur*, *Fmrp*), chromatin remodeling-related genes (*MeCP2*, *Foxp1*, *Ctnnb1*, *Pogz*, *Chd8*, *Chd10*), protein synthesis-related genes (*Tsc1/2*, *mTor*), and protein degradation-related gene (*Ube3a*). The functions of ASD-related genes in synapses and cells are shown in Figures 1C,D. However, due to inconsistencies of results in most genetic findings implicated in the pathogenesis of ASD, as well as edited genes’ differences between species, we summarized the examples of ASD models of single gene mutations from *Drosophila*, *zebrafish*, rodents, and non-human primates.

## *Drosophila* Models of ASD

*Drosophila* is used as an important model for ASD research because 75% of human disease-causing genes have functional fly homologs and flies have similar neural properties of the visual and motor systems as humans. *Drosophila* has a wide range of practical and genetic advantages, such as a short breeding time and a large number of offspring for rapid, large-scale analysis. These features allow efficient high-throughput genetic manipulation and greatly facilitate the discovery of single-gene function and high-level behavior.

A *Drosophila* fragile X syndrome (FXS) model was developed by using loss-of-function mutants or overexpression of the *Fmr1* homolog. In these models, poor synaptic structure was associated with perturbation of neurotransmission and synaptic type-specific alterations in both central and peripheral synapses (Zhang et al., 2001). The results showed that *dFmr1* (*Drosophila* fragile X mental retardation 1) mutant flies displayed considerable defects in the *Drosophila* stress odorant (dSO) response. Cyclic adenosine monophosphate (cAMP) signaling *via* PKA was activated after exposure to dSO and several drugs that regulated both cAMP and cyclic guanosine monophosphate (cGMP) levels significantly improved defects in dSO processing in *dFmr1* mutant flies (Androschuk et al., 2018).

Na^+^/H^+^ exchanger-3 (NHE3) controls the exchange of sodium and hydrogen ions in the cell membrane, which directly affects neural signaling. Reduced *Nhe3* expression affects the rate of sodium ion and proton exchange at the cell membrane. The separation between the first and second-order electro-physiological visual responses to homeostatic stimuli in *Nhe3* adult mutant flies is strikingly similar to response patterns in human adults with ASD (Vilidaite et al., 2018). *Drosophila* models phenotypically replicate FXS and *Fmr1* knockout mice, and pharmacological tests have been used to develop potential therapies. Altogether, these data reconfirm the suitability of *Drosophila* as a biomedical research model and its relevance to our understanding of genes and the physiopathology behind ASD.

## *Zebrafish* Models of ASD

*Zebrafish* is a good animal model for neural tube development, which has the advantages of *in vitro* development, embryonic transparency, and rapid development. It also has the main neural cell types including neurons, astrocytes, oligodendrocytes, and microglia. In *zebrafish*, common methods to create loss-of-function models include genomic knockout through mutagenesis and morpholino knockdown. Morpholino oligomers can be injected into early *zebrafish* embryos and transiently knock down the functional targeted genes by binding to the complementary target mRNA and blocking its translation in a manner similar to small interfering RNAs.

CRISPR/Cas9-induced *shank3* mutations, which caused ASD-like behaviors in *zebrafish*, resulted in reduced locomotor activity, and decreased levels of synaptic proteins. Additionally, reduced *shank3* expression in the *zebrafish* model system is responsible for gastrointestinal dysmotility, which resembles gastrointestinal symptoms commonly seen in patients with ASD (Liu C. X. et al., 2018; James et al., 2019). *Zebrafish* with *Cntnap2* mutations were overactive at night, and showed a GABAergic deficit, especially in the forebrain, and sensitivity to drug-induced seizures (Hoffman et al., 2016). A *Dyrk1aa* knock-out (KO) *zebrafish* was generated using TALEN-mediated genome editing. KO *zebrafish* showed social impairments consistent with human phenotypes of ASD (Kim et al., 2017).

Knockdown of *pten* (phosphatase and tensin homolog) by using antisense morpholino oligonucleotides (MOs) in *zebrafish* embryos inhibited convergent extension by affecting cell motility and protrusion during gastrulation. *pten* is an essential gene for proper control of cell movement and migration during *zebrafish* gastrulation, and regulates actin polymerization by antagonizing PI3 kinase and its downstream effectors AKT1 (AKT serine/threonine kinase 1) and CDC42 (cell division cycle 42; Yeh et al., 2011).

Based on the observation that *fmrp* knockdown *zebrafish* embryos exhibited increased anxiety, irritability, and cognitive impairment at 7 dpf (days post-fertilization), researchers concluded that this was an effective DNAzyme-based model for fragile X syndrome, which was associated with impaired craniofacial development and ASD-like behaviors (Medishetti et al., 2020). Although the CRISPR/Cas9 system is arguably the most commonly used gene-editing tool, other mutagenesis methods such as TALEN and ENU (N-ethyl-N-nitrosourea) are often used to create loss-of-function alleles in *zebrafish* studies. Henceforth, the study in the future should focus on the available genetic strategies applicable to zebrafish to develop reliable models to functionally validate ASD-candidate genes.

## Vertebrate Models of ASD-Related Syndromes

### Rodent models

Rodent models can recapitulate the molecular genetic defects associated with ASD patients and can serve as effective tools for dissecting the underlying molecular and cellular mechanisms of ASD. Rodent genetic models are developed based on well-studied monogenic or syndromic genes associated with ASD, such as *Shank3, MeCP2, Chd8, Pcdh10, Arid1b, Chd11, Chd9, Cntn4, Dip2a*, and *Casp3*. Reduced sociability and social agency presented in adult *Shank3* mutant mice are restored through gene re-expression. Lee’s results show that *Shank3* disruption can cause a decrease in neurons encoding the experience of other mice, while inducing an increase in neurons encoding their own experience. Recovery of *Shank3* expression reversed this coding imbalance and increased sociability within 5–8 weeks (Lee S. et al., 2021). This important finding points to gene therapy as a possible treatment for ASD.

It has been found that survival and phenotypic severity of male *MeCP2* null mice improved after neonatal intracranial delivery of single-stranded (ss) AAV9/chicken beta-actin (CBA)-MECP2 vector. Treated mice showed significant improvement in phenotypic severity of motor function and exploratory activity, and normalization of neuronal nuclear volume in transfected cells (Gadalla et al., 2013). In *MeCP2*-deficient mice, pyramidal neurons of the primary somatosensory cortex exhibited decreased spontaneous firing, decreased spontaneous excitatory synaptic input, and increased inhibitory drive, as well as decreased excitatory synaptic connectivity (Yu et al., 2020). The reversibility of the RTT-like phenotype in mice suggested that *MeCP2* gene substitution is a potential therapeutic option for patients. *Chd8*^+/–^ mice showed increased sensitivity to chemical injury and exhibited delayed dyskinesia, marked low activity, and abnormal ASD-like behavior to social stimuli, and increased synchronous activity in the cortico-hippocampus and auditory-parietal networks (Suetterlin et al., 2018). Female mice carrying a heterozygous mutation of *Chd8*^+/N2373K^ exhibited suppression of neuronal excitability, inhibitory synaptic transmission, and enhanced neuronal firing, as well as increased gene expression associated with extracellular vesicles and matrix (Jung et al., 2018). *Pcdh10*^+/–^ male mice showed increased dendritic spine density in immature morphology, while NMDAR expression was decreased, and displayed specific alterations in related behavior and mood (Ferri et al., 2021). Heterozygous *Arid1b* knockout (hKO) mice exhibit ASD-like traits associated with social behavior, anxiety, and persistence, as well as weight loss, impaired motor coordination, and hydrocephalus (Shibutani et al., 2017). *Chd11*-null mice, but not *Chd9*-null mice, have multiple autism-like behavioral changes (Wu N. et al., 2021). The small or missing regions of the anterior commissure of *Tbr1*^+/–^ mice lead to defective neuronal differentiation in the amygdala, which may serve as a suitable model for determining how autism genes control neuronal circuits, neural activity, and behaviors (Huang et al., 2019). The *Cntn4* KO mice model showed increased stress responses, decreased synaptic potentiation, and abnormal dendritic arborization and spines of hippocampal CA1 neurons (Oguro-Ando et al., 2021). *Dip2a* deficiency results in defective spine morphogenesis, PSD, and reduced synaptic transmission of pyramidal neurons, highlighting the contribution of synaptic protein acetylation to synaptic processing (Ma et al., 2019). Selective loss of the *Casp3* gene in the striatum by Cre-loxp-mediated recombination, significantly reduced potassium-induced dopamine release in mice. In addition, loss of *Casp3* can lead to impaired social interactions, restricted interests, and repetitive stereotypes in mice (Garcia-Dominguez et al., 2021). Although mice models are valuable for studying the pathogenesis of ASD, species-specific features in behavioral manifestation, as well as brain structure and functions pose a significant challenge to the use of small animals to simulate ASD in humans and translate experimental treatments to the clinic. Specifically, there are considerable differences between rodents and humans in many aspects, such as brain anatomy, cognitive capacity, and behavioral repertoire. Compared with rodents, non-human primates share great similarities with humans in key brain regions implicated in social behaviors.

### Non-human primate models

Rodent models have been widely used to model human genetic disorders, while the evolutionary differences between these two species have also been the focus of discussion regarding translational potentials. Non-human primates (NHPs) are considered to be better models for ASD than rodents because of their strong similarity to humans in terms of brain anatomy. Therefore, the use of NHPs to model human disease is of great importance, especially for neurodevelopmental disorders. Lentivirus-based transgenic cynomolgus monkeys overexpressing *MECP2* in the brain exhibited ASD-like behaviors, including a higher frequency of repetitive circular locomotion, increased stress responses, and fewer interactive behaviors. Interestingly, the cognitive functions of transgenic monkeys were essentially normal, even though some showed stereotypic cognitive behaviors (Liu et al., 2016). TALEN-edited *MeCP2* mutant cynomolgus monkeys showed similar phenotypes to ASD patients, including disruption of social interaction, social withdrawal, sleep disturbances, and reduced response to pain (Chen et al., 2017). Adeno-associated viruses (AAV)-delivered CRISPR/Cas9 gene editing can efficiently induce deletion of *MECP2* in the hippocampus of adolescent rhesus monkeys, and elicit core ASD-like phenotypes, including defects in social communication and interaction, hyposensitivity to sensory inputs, hypo-activity during the daytime, abnormal sleep patterns and hand motions (Wu S. et al., 2021). After CRISPR/Cas9-mediated disruption of *SHANK3*, mutant monkeys displayed core behavioral abnormalities, including impaired social interaction and repetitive behavior, apparent stereotypic movements, and reduced brain network activity detected by positron emission computed tomography (PET). Impaired social interaction and stereotypic behavior were significantly improved after fluoxetine treatment (Tu et al., 2019). Another study reported germline-transmissible mutations of *SHANK3* in cynomolgus macaques and their F1 offspring. The founder mutants displayed sleep disturbances, motor deficits, and increased repetitive behaviors, as well as social and learning impairment characteristic of ASD (Liu et al., 2019). Giant ankyrin 2 (*ANK2*), a strong candidate gene involved in nonsyndromic ASD, was depleted in cynomolgus and rhesus monkeys using CRISPR/Cas9 (Qin et al., 2021). Surprisingly, the depletion of *ANK2* in monkeys did not cause the core symptoms of ASD that had been observed in a mouse model (Yang et al., 2019). Rather, mutant monkeys showed typical abnormalities in brain development, including significant enlargement of lateral ventricles and loss of gray matter. This suggests that the functions of *ANK2* are evolutionarily divergent between rodents and primates. This finding provides further evidence that non-human primates are much better suited to recapitulate ASD-like behavior, which has great potential for the development of effective ASD treatments. The animal models of ASD are shown in Table 1.

### Relationship between rodent and NHPs ASD models

Rodent ASD models are faced with the challenges of relating the rodent brain to the human brain and rodent behaviors to human behaviors. Rodents are separated from humans by more than 70 million years of evolution (Gibbs et al., 2004). While, NHPs diverge from human evolution closer to 25 million years ago (Qin et al., 2019). In comparison to rodents, NHPs exhibit greater similarity to humans in genetic variation, brain development, brain size, functionally specialized brain structures and, ultimately, ASD-like behaviors. The brain regions implicated in social processing, such as the frontal, temporal, and anterior cingulate cortices as well as the amygdala, are either not well-developed or nonexistent in rodents, but are better developed in NHPs. Like humans, NHPs live in a complex social structure and have evolved a sophisticated social communication and interaction system, such as communicating with a variety of facial expressions, vocalizations, and body postures (Bauman and Schumann, 2018). However, reciprocal social behavior is limited in rodents, who rely heavily on olfactory communication. This is because not all brain regions implicated in ASD are well-developed in rodents, and genetic differences may also contribute to it. Undoubtedly, laboratory rats and mice provide ideal animal models for ASD research and comparative medicine studies due to their small size, ease of maintenance, short life cycle, and abundant genetic resources (Bryda, 2013). NHPs are ongoingly contributing to identify neural circuits and patterns related to behaviors affected by ASD. In the future, we should strengthen collaborative efforts between rodents and NHPs models to give full play to their advantages, and ultimately translate results from animal models into preventative strategies and/or novel therapies for human ASD.

Rodents have been widely used to study the pathogenesis of ASD, but it is not yet known whether we can accurately simulate and identify symptoms and mechanisms of ASD using rodent models. Non-human primates are not only related to humans in terms of genetic evolution, brain development, brain function, and social organization, but also exhibit very similar behavioral phenotypes. Cloning of macaque monkeys by somatic cell nucleus transfer (SCNT) can allow the generation of monkeys with uniform genetic backgrounds. This is very useful to expand ASD models with uniform genetic backgrounds and allows researchers to increase the significance level of the results (Liu Z. et al., 2018, Liu et al., 2019).

### Gene therapy

The monogenic aspect and severity of syndromic ASD make it an ideal candidate for gene therapy. The development of an antisense oligonucleotide (ASO) targeting toxic CGG repeats in FMR1 mRNA, reduced the number of pathogenic transcripts of FMR1 mRNA both *in vitro* and *in vivo* (Derbis et al., 2021). An ASO targeted to the native antisense transcript was able to un-silence the paternal allele and restore normal UBE3A expression *in vitro* and *in vivo* (Meng et al., 2015). The ASO targeting UBE3A-ATS was developed by two pharmaceutical companies and is currently in phase 1 or 2 clinical trials (Markati et al., 2021). Human hemopoietic stem cells were first transduced with an LV containing the UBE3A gene, and subsequently engrafted in the mouse. Eight weeks after birth, both newborn and adult mice showed normalized UBE3A levels, as well as phenotypic rescue (Adhikari et al., 2021). CRISPR/Cas9 has also been considered for the treatment of Angelman syndrome (AS) by knocking out the UBE3A antisense transcript, which can silence transcription. This strategy has been shown to be effective in AS mouse models and has ameliorated the disease phenotype (Wolter et al., 2020). Gene editing permanently changes the genome and can be a new therapy for ASD. Gene therapies with transient effects, such as ASOs, non-coding RNA, and RNA editing, which leave the genome unedited and require repeated administration, may have the advantage of being more controllable and reversible. These differences highlight important issues related to gene therapy, including dosing, administration, and safety (Weuring et al., 2021).

ZFN enables targeted homologous recombination in model organisms and human cells, which can edit single or multiple genes with high specificity. However, its application in gene therapy is significantly limited due to high off-target rate, slow speed, and high cost. TALEN is a highly specific single gene editing technology with a low off-target rate, short period, and weak cytotoxicity, but the cumbersome assembly process limits its application in ASD therapy. CRIPSR/Cas9 is not only used for genome editing but also adopted for transcriptional perturbation and epigenetic modulation. Whereas, the application of CRIPSR/Cas9 in ASD therapy is still a challenge due to low specificity. Although Cre/loxP provides cell- and time-specificity of gene editing, there is currently a limited application in ASD therapy.

## Future Perspective

Gene editing has the advantage of assessing gene interactions and the relationships between genes and phenotypes. ASD is a neurodevelopmental disease with considerable heterogeneity. Previous studies do not support the causality between the brain regions and ASD behaviors because of the apparent technical limitations in human studies. A variety of animal models designed to reproduce genetic or idiopathic ASD have highlighted potential therapeutic approaches targeting the core behaviors and underlying mechanisms of ASD. Gene re-expression with AAV vectors can reverse behavioral phenotypes or recover animal models from ASD symptoms, and these studies create a platform for clinical translation of various single gene forms of ASD. However, major obstacles remain pending issues, especially in most ASD disorders which are characterized by variable penetrance, epistasis, and a phenotype determined by the interaction between genetic and environmental factors. Furthermore, whether, or in what case, epigenetic factors can preclude reversibility, is still unclear. Future research should focus on improving the validity of animal models, also reliable DNA diagnostics and accurate prediction of the functional effects of the mutation will likely be equally crucial for the safe application of gene treatments.

## Author Contributions

All authors listed have made a substantial, direct and intellectual contribution to the work, and approved it for publication.
